# Delayed as-live surgery in Hospital Grand Rounds: How i do it

**DOI:** 10.1016/j.amsu.2021.102627

**Published:** 2021-07-27

**Authors:** Ronan A. Cahill

**Affiliations:** Department of Surgery, Mater Misericordiae University Hospital and Centre for Precision Surgery, School of Medicine, University College Dublin, Ireland

**Keywords:** Education, Grand rounds, Surgery, Operations, Video recordings, Delayed as live surgery

Hospital Grand Rounds are time-honoured, clinically-centred educational fora with their origins in Socratic-style beside teaching. They have evolved since their initiation in the late 19th century and are now most-often held in an auditorium with an audience of different levels of clinical experience and backgrounds comprising didactic presentations from in-house specialties or guest speakers usually with a focus on a disease or therapeutic process. While occupying a central part of the teaching timetable and academic budget resource (particularly for catering and invited speaker expenses), their relevance as general magazine in this era of supraspecialism has been questioned with many reporting dwindling attendances [1]. With particular regard to Surgical Grand Rounds, candid demonstration of the technical conduct of the operation itself is often under-represented during these sessions with line drawings, still photos and occasional video edits via PowerPoint being used as shorthand for the complex act of surgery. In particular, the critical aspect of real-time intraoperative decision-making is often hidden and the opportunity for engagement and shared learnings regarding different approaches to common technical components from different specialties is lost.

To help our faculty, residents and students engage more with the core matter of operative surgery at Grand Rounds, we have successfully introduced delayed as-live operating episodes within our weekly surgical grand rounds that needs to appeal to team members of 12 different surgical specialties including undergraduates [2]. We have found these sessions enable immersion of the audience in the operative aspects of the topic at hand, promote candid discussion and cross-specialty interaction (especially with the inclusion of intraoperative complications and unexpected findings) and allow showcase of faculty as operating clinicians alongside their teaching and research roles. While elegant technological services exist to provide outstanding video delivery [3], our rather basic set-up is here described.

When constructing the session, showcasing three operations works best within our routine 45-min session with all fitting a theme either related to specialty (e.g. lower gastrointestinal surgery or urology), access/technology (e.g. robotics) or complication (e.g. hemorrhage or visceral perforation). Videos are recorded from recent operations performed in the hospital (thus the procedure is fresh in the presenter's mind) with patient consent re their use for teaching and the recordings screened for any patient identifying details. Minimally invasive approaches suit the format well as the screen display of the procedure can be easily recorded and, when replayed, the video shares exactly the view of the operating team. Three unedited core segments of approximately 7 min duration are chosen from each operation and made into separate clips via simple cropping using basic video management software (for an example see Table). The video segments are labelled after the Theatre most associated with the specialty (in our case Theatre 10 is our gastrointestinal theatre and Theatre 6 is our dedicated emergency theatre) and are set up on the podium computer in multiple open windows using VLC Video player. This free downloadable software thereby allows each video be ready for playing from a thumbnail view viewable only to the session chair. During the session, the videos clips from each operation are shown one at a time, (with the other video segments open but minimized and ready to play) with the session chair moving between the clips and so the operations in an ordered manner (see Table 1) as if the three surgeries are being performed simultaneously with live narration transmitted to the auditorium via a microphone from the surgeons who performed the cases from outside the room. Although overall operative timeframes are compressed to fit the session, the movement between cases allow key steps from each be shown without breaking audience engagement with the topics on display and while maintaining a sense of operative step-sequence continuity. The narrating surgeons are best positioned outside of the room to encourage audience immersion and ‘suspension of disbelief’ in the cases so they engage as if they are being performed live (each narrator should describe the operative steps as if they are doing the procedure live including silence during any step needing focus).

**Table 1:** Set-up of video clips including video segment running order (1–9).

Case	Clip One	Clip Two	Clip Three
Laparoscopic Cholecystectomy	Trocar insertion [1]	Display of Calot's triangle [4]	Dissection of Gallbladder from liver bed (+/− bile leak)(7)
Laparoscopic Appendicectomy	Initial view of pelvis & appendix [2]	Mesoappendix dissection (+/− bleeding) [5]	Endolooping and division of appendix base (8)
Laparoscopic Right Hemicolectomy	Identification of ileocolic artery [3]	Proximal or Distal Stapled Transection [6]	Intracorporeal anastomosis (9)

The key to the audience's engagement is confident compering by the session chair and credible narration from and interaction with the surgeon detailing the operative steps at any one time which is greatly helped by a practice run-through in advance. The chairperson needs to both interact with the narrator and the audience showing that comments and questions can be transferred to the narrator and discussion on topics of general interest among specialties can be shared. The narrators each need be comfortable with comment on and alternative perspectives of their component steps and be capable of discussion of nuanced points while also being embedded in the part of the operation being displayed. To help this, they need to be able to view the segment playing in real-time and, ideally, be able to hear the audience comments (positioning just outside the auditorium door with a view of the main auditorium screen through a glass panel facilitates this). The session chair need be mindful of time and be able to switch to another clip before the segment ends but also be sensitive enough to the audience's interest not to move too soon if the discussion is proving useful. We find that 5 min per clip is sufficient to demonstrate a key operative step and get a measure of the case momentum with two extra minutes being available for extension given particular interest in the operative step. The combination of live, engaged voices from the chair and the different narrators interacting with audience comments proves a compelling session with many in the audience quickly suspending their disbelief and imagining the operations as indeed live.

The concept of delayed live operating is not ours, I witnessed it for the first time at the European Association of Endoscopic Surgery where it was masterfully delivered by Professor Jaap Bonjer of the Amsterdam University Medical Centre with an outstanding faculty and a technological team of broadcast quality expertise and equipment. Its background is in the concern regarding transmission of live surgery potentially impacting surgical quality [4] (live operating is anyway incompatible with grand rounds timing and lacks strong evidence of benefit [5,6]). These grand round sessions have become among the most favourably appreciated by participants and audience members especially students based on formal feedback and engender considerable discussion throughout the rest of the day.

## Ethical approval

Not needed.

## Sources of funding

None.

## Author contribution

Single author.

## Registration of research studies not applicable

1. Name of the registry:
2. Unique Identifying number or registration ID:
3. Hyperlink to your specific registration (must be publicly accessible and will be checked):

## Guarantor

Ronan Cahill.

## Consent

Not applicable.

## Declaration of competing interest

None related to this work. Re research, Dr Cahill has received speaker payments from Medtronic, Ethicon, Olympus and Stryker consulting payments from Medtronic and Distalmotion and holds research funding with Palliare (EU via Horizon 2020) and IBM Research and Deciphex (Irish Government via DTIF, Enterprise Ireland).
